# An Italy-China Collaboration for Promoting Public Mental Health Recommendations During the COVID-19 Pandemic

**DOI:** 10.3389/fpubh.2021.640205

**Published:** 2021-08-13

**Authors:** Maria Luisa Scattoni, Fabrizio Starace, Giovanni de Girolamo, Jun Xia

**Affiliations:** ^1^Istituto Superiore di Sanità, Research Coordination and Support Service, Rome, Italy; ^2^AUSL Modena, Department of Mental Health and Drug Abuse, Modena, Italy; ^3^Italian Society of Epidemiological Psychiatry (SIEP), Modena, Italy; ^4^Saint John of God Clinical Research Center, Psychiatric Epidemiology and Evaluation Unit, Brescia, Italy; ^5^University of Nottingham Ningbo, Nottingham Ningbo GRADE Centre, Nottingham, China

**Keywords:** COVID-19, mental health, health policies, international collaboration, Italian guidelines

## Abstract

The rapid evolution of severe acute respiratory syndrome coronavirus 2 (SARS-CoV-2) emergency involved Italy as the first European country. Meanwhile, China was the only other country to experience the emergency scenario, implementing public health recommendations and raising concerns about the mental health of the population. The Italian National Institute of Health [Istituto Superiore di Sanità (ISS)] reviewed relevant scientific literature in mental health to evaluate the best clinical practices and established the collaboration with the WHO, World Psychiatry Association, and China to support the public health system in a phase of acute emergency. This process permitted the definition of organizational and practical-operational Italian guidelines for the protection of the well-being of healthcare workers. These guidelines have been extensively disseminated within the Italian territory for maximum stakeholder utilization.

## Introduction

### The Rapid Evolution of the Italian SARS-CoV-2 Emergency

Starting from February 2020, Italy was the first European country facing the severe acute respiratory syndrome coronavirus 2 (SARS-CoV-2) emergency. On March 8, 2020, several Northern Provinces of Italy were placed under lockdown, and on March 10, 2020, at 00:30, any movement of people across the whole Italian national territory was forbidden, unless motivated by compelling reasons of health or work. All Italian people suddenly experienced confinement at home, physical distancing, while simultaneously receiving dramatic news from all media concerning the pandemic evolution and hospital emergencies, with potential negative impacts on the mental health of the people, as shown in a recent German study (1).

The transmission of SARS-CoV-2 was particularly rapid and dramatic in northern Italy, where COVID-19 health workers were suddenly asked to implement changes in activities and processes of care, such as team and workspace procedures. Frontline health workers were, and continue to be among the worst affected, facing shortages of personal protective equipment and enduring exhausting work schedules (2).

At the peak of the Italian pandemic, China was the only other country to experience the emergency scenario and lockdown. At that time, China had already implemented a series of public health recommendations and raised concerns about the mental health of the general population and high-risk groups (3).

### The Section on Policy Options and Implications

#### Concerns for Mental Health Problems

As a consequence of the lockdown, Italian people experienced a sharp change in their behavior as well as living and working environment. Prolonged confinement, change of routines, lack of economic resources, and uncertainty of lockdown duration, caused several adverse psychological effects such as stress, anxiety, depression, and frustration in the general population (4). Evidence suggested that women were more often reporting anxiety symptoms. Indeed, women were almost three times more likely than men to report anxiety symptoms during the COVID-19 pandemic (5, 6). Moreover, the school closure and quarantine have placed considerable psychological stress on children and adolescents that were unable to maintain their interpersonal life and their free time activities and sports (7). Social media and social networks could be used as a tool for online counseling, restoring daily routines, and communication. However, social media activities should be considered with caution, as the length and frequency of media consumption is a significant risk factor associated with increased anxiety and depression (8, 9). Moreover, susceptibility to mental health problems was compounded by the experience of dramatic life events, such as bereavement, unavailability of final farewell in terminal situations, and parental separation due to confirmed or suspected infection. Another important behavioral change caused by the lockdown and the parallel closure of many outpatients mental health services and day-centers was represented by the sudden increase in the amount of time spent face-to-face between a patient and his/her family members, increasing the risk of relapse, higher levels of symptoms, and poorer functioning in patients with psychotic disorders (10).

Public health services received detailed instructions on overcoming challenges in providing mental healthcare (11), especially for individuals with existing mental health conditions, who were at high risk of negative outcomes (12–15). For example, efforts were made to provide telehealth interventions, which helped some, but proved challenging for individuals with higher needs of support, such as people with autism spectrum disorder and intellectual disability. While telehealth has been advocated as an effective strategy to keep contacts with patients in treatment, organize a triage of new requests, respond to requests of changes in medication profiles, and set up videoconferencing with patients needing psychological support and counseling, it also raises several problems (e.g., effectiveness, quality, and therapeutic relationship established with a patient) which should be carefully investigated (16).

The Italian National Institute of Health [Istituto Superiore di Sanità (ISS)] consequently elaborated specific guidelines for COVID-19 diagnostic and care procedures, particularly for individuals in the autistic spectrum and/or with intellectual disabilities, to optimize the management of sharp changes in the living and working environment and daily routine of such individuals. The main areas addressed by the ISS guidelines included strategies to promote the training of professionals, reorganize clinical and care activities through the development and maintenance of remote interventions, scheduled interventions in the presence, and patient management in the long-term facilities.

Finally, frontline health workers found themselves exposed to biological risk factors, social isolation from family members, and subsequently, intensified challenges of recovery from physical and emotional distress (as shown in Table 1). Moreover, in COVID-19 care settings, they experienced a lack of effective interventions and changes in the ways of communicating with the families of patients (17). Overburdening and prolonged stress affects attention focus comprehension and decision-making, which can have a lasting effect on overall well-being (11). Therefore, the protection of health professionals and the promotion of their health and well-being are among the key components of public health measures in tackling the COVID-19 epidemic (11, 18–22).

**Table 1:** Physical and emotional challenging factors of health professional workers as a consequence of the rapid evolution of the severe acute respiratory syndrome coronavirus 2 (SARS-Cov-2) emergency {Italian National Institute of Health [Istituto Superiore di Sanità (ISS)] Working Group on Mental Health and Emergency COVID-19, 2020}.

**Challenging factors**	**Description**
**Physical**	Biological risk
	Difficulty in finding individual protection devices
	Excessive workload
	Change teams and workspaces/places
	Progressive updating of activities and procedures
	Lack of rest
**Emotional**	Management of complex patients
	Lack of treatment of proven effectiveness
	High responsibility
	The burden of expectations and fear of not doing enough
	Feelings of vulnerability or loss of control
	Lack of contact or prolonged interruption of relationships with family members
	Concern about their health and spreading the infection to their family members

### Actionable Recommendations

#### Learning From an International Experience: the Italy-China Collaboration to Strengthen Public Health Policies

The ISS is the main Italian center for research, control, and technical-scientific advice on public health and health guidance policies, as informed by scientific evidence alongside the Ministry of Health, the Regions, and the entire National Health Service. The ISS, in this as in other situations, cooperates with experts from other international organizations (e.g., WHO, UNICEF, Save The Children, and World Psychiatry Association). Only China, at that time, was devoted to face the COVID-19 pandemic and its consequences on the mental health of the people. The ISS researchers reviewed relevant scientific literature in mental health to evaluate the best clinical practices. We established the collaboration with the World Psychiatric Association Action Plan 2021–23 Working Groups on Intellectual Developmental Disorder and Autism Spectrum Disorder, consolidating a working group that formulated advice to tackle mental distress for people with intellectual disability and autism spectrum disorder, SIDiN (23). We also identified the Chinese guideline entitled “Guideline for Psychological Adjustment During the COVID-19 Pandemic” produced by the Disease Control and Prevention Department, National Health Commission of the People's Republic of China. This document offered psychological adjustment guidance for the general population and specific at-risk groups (e.g., elderly, children, teenagers, pregnant women, people in home isolation, confirmed and suspected patients with COVID-19 and their relatives, and relatives of people dying of COVID-19), such as frontline workers for COVID-19. In addition, the guidelines described strategies for the adjustment of different psychological problems such as sleep problems, anxiety, panic, and worries about being infected.

The Research Coordination and Support Service of the ISS collaborated with the Nottingham Ningbo GRADE Center and researchers of the Shanghai University of Traditional Chinese Medicine, and Children's Hospital of Fudan University to translate and adapt the aforementioned guidelines for an Italian context, with consideration of contextual and cultural differences. The translation of Chinese guidelines has been useful in the preparation of the organizational and practical-operational guidelines for the health workers report “Interim guidance for the appropriate support of the health workers in the SARS-CoV-2 emergency scenario,” developed by the ISS Mental Health and Emergency Working Group COVID-19, which is accessible on the institutional ISS website (https://www.iss.it/en/rapporti-iss-covid-19-in-english/). This ISS report is freely available and has been extensively disseminated within the Italian territory for maximum stakeholder utilization. The report provides guidelines to organize the roles and activities of workers, ensure training (e.g., distance training, technical reports), promote material in support of interventions, and monitor psychological well-being such as individual support strategies, monitoring of reactions related to discomfort, activate psychological and psychiatry support, psychological, psychiatric, and psychopharmacological interventions (as shown in Table 2).

**Table 2:** The management of physical and emotional challenging factors of health professional workers during the SARS-Cov-2 emergency scenario (ISS Working Group on Mental Health and Emergency COVID-19, 2020).

**Organizational and practical-operational guidelines**
**Organization of workers' roles and activities**	Coordinate communication
	Organize workspace and time
	Encourage sharing and teamwork
	Favor homogeneous practices between operative units
	Recognize and value the personal and professional contributions
**Ensuring training**	Distance learning
	Technical reports of institutional websites
	Documents and resources of scientific societies
**Promoting material support interventions**	Availability of Individual Protection Devices
	Provision of dedicated of resting places in the working environment
	Supply the basic necessities (e.g. food)
	Support in the management of children
	Provide economic recognition
**Promoting individual support strategies**	Promote nutrition, sleep and exercise
	Avoid stress and encourage emotional support
	Encourage activities and working group
	Encourage correct information
**Monitoring reactions related to discomfort**	Food and sleep
	Fatigue and physical symptoms
	Tension and psychological symptoms
	Stress management behaviors

## Conclusions

The collaboration between Italy and China, established in a phase of acute emergency, has been fundamental as a basis for the definition of Italian guidelines dedicated to public mental health and promotion of the well-being of health workers, whose protection is a key component of public health measures in addressing the COVID-19 epidemic (15). The process was supported by consultation with the World Psychiatry Association and mental health guidelines were disseminated *via* an e-meeting organized by the WHO working in Ethiopia on Mental Health and COVID-19. This document describes an evidence-based process that can be taken as an example for future international collaborations to support the public health systems.

## Author Contributions

MLS: conceptualization and funding acquisition. MLS, FS, GG, and JX: writing the original draft preparation, writing the review and editing. All authors have read and agreed to the published version of the manuscript.

## Conflict of Interest

The authors declare that the research was conducted in the absence of any commercial or financial relationships that could be construed as a potential conflict of interest.

## Publisher's Note

All claims expressed in this article are solely those of the authors and do not necessarily represent those of their affiliated organizations, or those of the publisher, the editors and the reviewers. Any product that may be evaluated in this article, or claim that may be made by its manufacturer, is not guaranteed or endorsed by the publisher.
